# Hepatotoxicity associated with statins: A retrospective pharmacovigilance study based on the FAERS database

**DOI:** 10.1371/journal.pone.0327500

**Published:** 2025-07-09

**Authors:** Lu Zhou, Bin Wu, Yuan Bian, Yun Lu, Ya Zou, Shanshan Lin, Qinchuan Li, Chun Liu

**Affiliations:** 1 Department of Pharmacy, Chengdu Second People’s Hospital, Chengdu, Sichuan, China; 2 Department of Pharmacy, West China Hospital, Sichuan University, Chengdu, Sichuan, China; 3 Department of Pharmacy, Sichuan Provincial People’s Hospital, University of Electronic Science and Technology of China, Chengdu, China; 4 Department of Pharmacy, The Second Affiliated Hospital of Fujian Medical University, Quanzhou, China; Christian Medical College Vellore, INDIA

## Abstract

**Background:**

Statins are commonly prescribed in clinical practice and are associated with a high risk of drug-induced liver injury (DILI). This study aims to examine the real-world data on statin-induced liver injury to assess medication safety.

**Methods:**

All DILI cases reported with statins as primary suspected drugs were extracted based on the US Food and Drug Administration adverse event reporting system (FAERS) from 2004 to 2023. A disproportional analysis was conducted using reported odds ratios (ROR) and information component (IC) to assess the significant association between statins and DILI.

**Results:**

A total of 7779 statin-associated DILI cases were identified. DILI patients tended to be aged >65 years (45.43%), with more females than males (48.80% vs 43.75%), and 39.95% of DILI patients required hospitalization. Statin-induced DILI cases are most commonly reported with atorvastatin (53.48%), rosuvastatin (20.44%), and simvastatin (19.46%). The DILI signals (ROR; 95% CI) for statins were ranked as follows: fluvastatin (6.90; 5.89–8.10)> atorvastatin (3.09; 2.99–3.19)> simvastatin (2.96; 2.81–3.12)> lovastatin (2.77; 2.17–3.53)> rosuvastatin (2.27; 2.16–2.39)> pravastatin (2.07; 1.81–2.37). Age-stratified analysis showed that a stronger signal was detected in patients (aged ≥65 years) than patients (aged <65 years) for atorvastatin, simvastatin, pravastatin and fluvastatin. The onset time of DILI was significantly different among the different statins (*p* = 0.014), and simvastatin resulted in the highest mortality rate (12.15%).

**Conclusion:**

Based on FAERS database, six statins are significantly associated with liver injury, and fluvastatin, atorvastatin, and simvastatin had the greatest risk of DILI.

## Introduction

Drug-induced liver injury (DILI) is a global health concern due to its prevalence and potentially life-threatening consequences [1,2]. The symptoms of DILI range widely, from mild elevations in liver enzymes to severe liver conditions such as cirrhosis or acute liver failure (ALF) [3]. DILI accounts for approximately 10% of all acute hepatitis cases, causes acute jaundice in nearly 50% of new-onset jaundice patients, while being increasingly acknowledged as the leading cause of ALF, particularly in regions with low viral hepatitis (e.g., HBV, HCV) prevalence [4–7]. According to reports, at least 1,000 drugs can cause liver injury, with detailed information available on specialized websites such as LiverTox (www.livertox.org) and Hepatox (www.hepatox.org) [8,9].

Due to variations in prescribing and medication practices, as well as population heterogeneity, the prevalence and etiology of DILI exhibit regional differences [10]. According to epidemiological surveys, the estimated incidence of DILI in the United States, England, France, and Iceland is approximately 2.4, 2.7, 13.9 and 19 cases per 100,000 patients, respectively [11–14]. In China, DILI has occurred more frequently, reaching 23.9/100,000 [15]. Hypolipidemic drugs, including statins are among commonly reported to be associated with hepatotoxicity [3]. A Swedish study reported that DILI accounted for 57% of adverse reactions attributed to statins between 1988 and 2010 [16].

Cardiovascular disease (CVD) is one of the most common causes of premature death globally, with disorders of lipid metabolism being a primary risk factor for CVD development [17–19]. Statins, 3-hydroxy-3-methylglutaryl coenzyme A (HMG-CoA) reductase inhibitors, represent a cornerstone therapy for lowering blood cholesterol levels and are recommended as essential agents for both primary and secondary prevention of CVD [20]. With the aging of the global population and the increasing burden of CVD, the clinical reliance on statins has grown substantially. For example, the number of annual statin prescriptions in the United States exceeded 30 million, and those in England exceeded 61 million [21]. In China, atorvastatin alone sold 3.78 billion RMB, far ahead of the use of other lipid-lowering drugs in 2022 [22].

Given such widespread use and long-term consumption, it is critical to summarize the potential risks and benefits of statins. Some retrospective and prospective series, case reports and review involving DILI have reported statin-induced hepatotoxicity [13,23–26]. However, the relationship between statins and liver dysfunction remains controversial, and few large real-world sample studies available [23,27,28]. For example, statins are listed as one of the causes of ALF by the Acute Liver Failure Study Group in the US [23]. Conversely, another study indicated that persistent elevations in transaminases exceeding three times the upper limit of normal due to statin therapy are frequently observed but rarely associated with hepatic adverse events [28].

The FDA’s Adverse Event Reporting System (FAERS) is one of the largest open pharmacovigilance databases that can be used to evaluate and identify adverse drug reactions [29]. Thus, the objective of this study was to detect and analyze the signals of statin-induced hepatotoxicity in the FAERS, identify potential high-risk safety signals, and compare the characteristics of hepatotoxicity caused by different statins in a large population. This study further provided a reference for the rational and safe clinical application of statins.

## Methods

### Data source

This retrospective pharmacovigilance study was an analysis of adverse drug event cases obtained from the FAERS database. The FAERS data contains patient demographic and administrative (DEMO), drug information (DRUG), report sources (RPSR), adverse events (REAC), patient outcomes (OUTC), indications (INDI), and therapy start and end dates for the reported drugs (THER) [30]. All the data can be accessed at https://fis.fda.gov/extensions/FPD-QDE-FAERS/FPD-QDE-FAERS.html. Definitions for FAERS database fields can be found on the FDA official website. Specifically, definitions for patient outcomes (OUTC)—such as Disability (DS) and Congenital Anomaly(CA) —are standardized by FDA criteria (available at https://www.fda.gov/safety/reporting-serious-problems-fda/what-serious-adverse-event).

The data from the first quarter of 2004 to the fourth quarter of 2023 were retrieved from the FAERS Quarterly Data Extract Files, and further managed using the SQL server 2017 software for analysis. Based on the FDA recommendation, the original datasets were deduplicated and standardized by choosing records with the latest FDA_DT and removing the same CASE number (CASEID) [31]. If the ‘FDA_DT’ and ‘CASEID’ were the same, the higher ‘PRIMARYID’ was selected.

### Target drugs and DILI identification

In the DRUG table, drug names can be recorded in various forms, such as generic names, synonymous names, trade names, or abbreviations. Before pinpointing the target drugs, we utilized MedEx software to standardize different medication forms into “generic names” for the purpose of obtaining statin-associated cases. Obtaining statin-associated cases: MedEx software was used to standardize different medication forms into a “generic name” [31,32]. We attempted to identify eight statins as classified by the World Health Organization (WHO) Anatomical Therapeutic Chemical (ATC) Classification System in the DEMO and DRUG table as follows: atorvastatin (C10AA05), cerivastatin (C10AA06), fluvastatin (C10AA04), lovastatin (C10AA02), pitavastatin (C10AA08), pravastatin (C10AA03), rosuvastatin (C10AA07) and simvastatin (C10AA01). Furthermore, the role of the drug in the DRUG file was restricted to ‘primary suspected (PS)’.

The adverse events are recorded in the REAC table, mainly coded using the Medical Dictionary for Regulatory Activities (MedDRA) (Version 25.1) Preferred Terms (PTs). DILI cases were identified in two ways. We used the Standardized MedDRA Queries (SMQs) coded 20000007 narrow searching to investigated DILI adverse events, including 144 PTs (S1 Table). For cases involving several PTs of the same SMQ, only one record was retained after duplicates were eliminated. Additionally, we excluded the cases in which the start date of drug treatment was later than the event date of the DEMO table. Specifically, those cases related to liver injury events that occurred prior to the start of statin treatment were removed from our analysis.

### Data mining

Based on Bayesian analysis and disproportionality analysis, we investigated the association between statins and DILI by employing the reporting odds ratio (ROR) and the information component (IC) as metrics to quantify the association’s strength [22,33]. We conducted a comprehensive enumeration of the characteristics pertaining to DILI events with statins, encompassing cases attributed to different statins, gender, age, reporter type, reporting country, the median time-to-onset of hepatotoxicity, and case outcomes.

For ROR, a signal was detected when there were at least 3 reported cases and the lower limit of the 95% confidence interval (95% CI) exceeded 1. For the IC, a signal was detected when the IC exceeded 1 and the lower limit of the 95% CI was greater than 0. The methodologies employed for these calculations are detailed in S2 Table [34,35]. An incident of DILI was classified as statin-associated if it satisfied the criteria for both analytical algorithms as described above.

Statins are the main drugs used for the primary and secondary prevention and treatment of cardiovascular and cerebrovascular diseases, and the population used is mainly the elderly population. To reduce age-related confounding effects, we conducted an age-stratified analysis.

### Statistical analysis

The characteristics of statin-induced DILI cases from FAERS were summarized by descriptive statistics. Continuous variables were reported as median (interquartile range [IQR]); categorical variables were presented as numbers and percentages (%). The chi-square test was utilized to compare categorical variables, including differences in the demographic characteristics such as patient age, sex group, type of reporter and region of occurrence, outcomes between DILI and non-DILI cases; differences in occurrence region, hospitalization rate and mortality among different statins; as well as differences in statin dosing. One-way ANOVA analysis was used to compare the time to onset of DILI caused by various statins which were reported as the median. It was regarded as statistically significant if P < 0.05. All analyses were conducted by the software GraphPad Prism 9, Microsoft Excel 2016 and SPSS version 26.0. The reporting of this study conforms to the Strengthening the Reporting of a Disproportionality Analysis for Drug Safety (READUS) statement [36].

## Results

### Identification of DILI from FAERS

After filtering, 167,328 cases associated with primary suspected statins were retrieved from the FAERS database. We excluded 216 cases with pre-existing liver injury complications. Finally, 167,112 adverse reactions cases were included, including 7,779 cases reported with DILI events (DILI group) and 159,333 cases reported with other adverse events (non-DILI group). The following eight statins were identified: atorvastatin (4,160 cases, 53.48%), rosuvastatin (1,590 cases, 20.44%), simvastatin (1,514 cases, 19.46%), pravastatin (220 cases, 2.83%), fluvastatin (169 cases, 2.17%), lovastatin (68 cases, 0.87%), pitavastatin (52 cases, 0.67%), and cerivastatin (6 cases, 0.08%).

**Fig 1:**
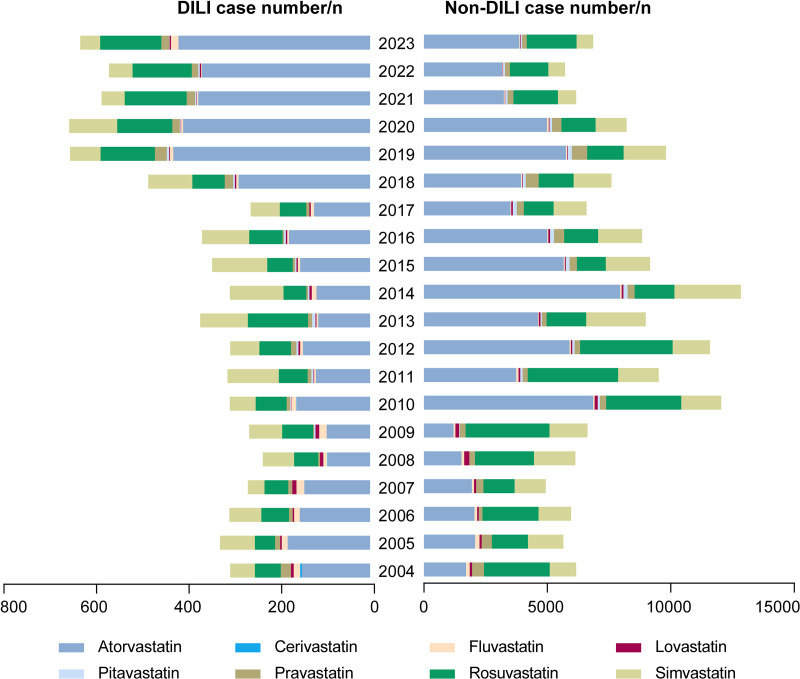
Annual number of reported DILI and Non-DILI cases associated with statins in FAERS.

The number of reported statin-related DILI cases use remained almost stable each year from 2004 to 2017. The percentages of these cases showed an upward trend after 2017, especially as the number of reports of atorvastatin and rosuvastatin related DILI almost doubled (Fig 1).

### Descriptive analysis

Overall, among the 7,779 statin-related DILI cases, atorvastatin, rosuvastatin, and simvastatin were the main contributors, accounting for 53.48%, 20.44%, and 19.46%, respectively, significantly higher than other statins. The detailed clinical features of the statin-associated AE cases were summarized in Tables 1 and S3–S13. DILI cases predominantly occurred in the elderly patients (≥65 years) group (3,534 cases, 45.43%). Notably, among the rosuvastatin cases, the younger group (<65 years) was more prevalent than the elderly group (41.82% vs. 35.72%). The female-to-male ratio of all statin-related DILI cases was 1.15. Statins with a slightly higher DILI rate in females were fluvastatin(63.31% vs. 32.54%), followed by atorvastatin (51.25% vs. 41.75%), and pravastatin (50.91% vs. 40.00%). Health professionals reported the vast majority of DILI cases and non-DILI cases (76.96% vs. 45.49%). In addition, as the daily dosage of atorvastatin increased incrementally from 10 mg/d to 80 mg/d, the number of DILI cases correspondingly rose from 471 to 655, demonstrating a upward trend (P <0.001, S11 Table).

**Table 1:** Demographic characteristics of DILI cases associated with statins and non-DILI cases in the FAERS.

Characteristics	DILI cases		Non-DILI cases	
	Case number (n)	Proportion (%)	Case number (n)	Proportion (%)
***Statin cases***				
Atorvastatin	4160	53.48	78562	49.31
Rosuvastatin	1590	20.44	40501	25.42
Simvastatin	1514	19.46	29670	18.62
Pravastatin	220	2.83	6142	3.85
Fluvastatin	169	2.17	1415	0.89
Lovastatin	68	0.87	1419	0.89
Pitavastatin	52	0.67	1598	1.00
Cerivastatin	6	0.08	26	0.02
***Age group***				
< 65 years	2821	36.26	50935	31.97
≥ 65 years	3534	45.43	57115	35.85
Unknown	1424	18.31	51283	32.19
***Gender***				
Female	3796	48.80	75764	47.55
Male	3403	43.75	67113	42.12
Unknown	580	7.46	16456	10.33
***Outcomes***				
Death	679	8.73	7220	4.53
Life threatening	587	7.55	5556	3.49
Hospitalization	3108	39.95	28401	17.82
Disability	144	1.85	8561	5.37
Congenital anomaly	2	0.03	212	0.13
Required intervention	45	0.58	1285	0.81
Other serious events	2852	36.66	64252	40.33
Unknown	362	4.65	43846	27.52
***Reporter occupation***				
Health-professional	5987	76.96	72486	45.49
Non-health professionals	1274	16.38	70416	44.19
Unknown	518	6.66	16431	10.31
***Reporter region***				
North America	2165	27.83	91249	57.27
Europe	3818	49.08	42672	26.78
Asian	1000	12.86	6552	4.11
Oceania	105	1.35	1872	1.17
South America	116	1.49	5913	3.71
Africa	33	0.42	620	0.39
Unspecified	542	6.97	10455	6.56

In terms of reporter region, the most frequent DILI cases were reported from Europe (3,818 cases, 49.08%), followed by North America(2,165 cases, 27.83%), Asian (1,000 cases, 12.86%), South America (116 cases, 1.49%), Oceania (105 cases, 1.35%), and Africa (33 cases, 0.42%). Notably, regional reporting disparities were observed across different statins. DILI cases associated with rosuvastatin and lovastatin predominantly originated from North America (624 cases, 39.25% and 45 cases, 66.18%, respectively), while atorvastatin, simvastatin, and pravastatin showed a European predominance (2,300 cases, 55.29%; 845 cases, 55.81%; and 126 cases, 57.27%, respectively), and fluvastatin and pitavastatin cases were predominantly reported from Asia (73 cases, 43.20% and 28 cases, 53.85%, respectively), with significant interregional differences across statins (P < 0.001, S9 Table). Additionally, there was significant difference in patient age, sex group, type of reporter and reporter region between DILI and non-DILI cases (*p* < 0.001).

### Patient outcome analysis

The rates of mortality, life-threatening, or hospitalization due to statin-induced liver injury events were assessed to analyze the patient prognosis. The results of OUTC are shown in Tables 1 and S11–S13. Compared with the non-DILI group, patients in the DILI group tended to have a poorer prognosis. of DILI-related outcomes in primary suspected statins, hospitalization, life threatening and death accounted for 39.95% (3,108 cases), 7.55% (587 cases) and 8.73% (679 cases), respectively. The hospitalization rate for pitavastatin-related DILI ranked first among the eight drugs, followed by pravastatin, atorvastatin, simvastatin and fluvastatin, and the differences between different statins were significant (*p* < 0.001). The mortality rate of simvastatin was seemingly the highest (184 cases,12.15%), whereas the mortality rate of cerivastatin was the lowest (0 cases, 0.00%). Additionally, no statistically significant difference was observed in mortality risk across different doses of atorvastatin (*P* = 0.123), while hospitalization rates all exceeded 39% (*P* = 0.001).

### DILI signal detection

We first applied ROR and IC to analyzed the data based on statins. Despite we attempted to detect the signals of eight suspected statins, only significant signals for six statins were detected: fluvastatin (ROR: 6.90, 95% CI: 5.89–8.10; IC: 2.65, 95% CI: 2.08–3.13), atorvastatin (ROR: 3.09, 95% CI: 2.99–3.19; IC: 1.56, 95% CI: 1.46–1.67), simvastatin (ROR: 2.96, 95% CI: 2.81–3.12; IC: 1.51, 95% CI: 1.34–1.68), lovastatin (ROR: 2.77, 95% CI: 2.17–3.53; IC: 1.43, 95% CI: 0.59–2.19), rosuvastatin (ROR: 2.27, 95% CI: 2.16–2.39; IC: 1.15, 95% CI: 0.98–1.32), and pravastatin (ROR: 2.07, 95% CI: 1.81–2.37; IC: 1.02, 95% CI: 0.57–1.46), respectively (Table 2). No positive signals were detected only for cerivastatin and pitavastatin.

**Table 2:** Signal detection of statin-associated DILI cases in the FAERS.

Drug/PT	DILI cases (n)	All AE cases (n)	ROR	95% CI for ROR	IC	95% CI for IC
Atorvastatin*	4160	78562	3.09	2.99 to 3.19	1.56	1.46 to 1.67
Rosuvastatin*	1590	40501	2.27	2.16 to 2.39	1.15	0.98 to 1.32
Simvastatin*	1514	29670	2.96	2.81 to 3.12	1.51	1.34 to 1.68
Pravastatin*	220	6142	2.07	1.81 to 2.37	1.02	0.57 to 1.46
Fluvastatin*	169	1415	6.90	5.89 to 8.10	2.65	2.08 to 3.13
Lovastatin*	68	1419	2.77	2.17 to 3.53	1.43	0.59 to 2.19
Pitavastatin	52	1598	1.88	1.43 to 2.48	0.89	−0.04 to 1.77
Cerivastatin	6	26	13.33	5.49 to 32.49	3.46	−0.54 to 4.87

### Age stratified analysis

**Fig 2:**
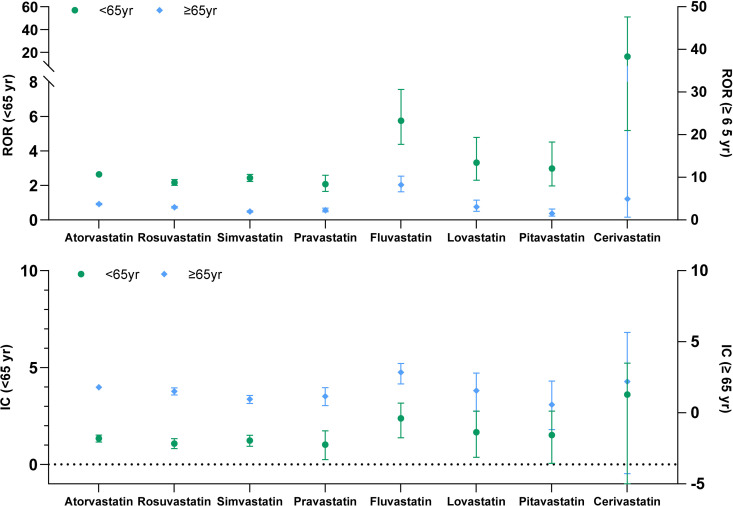
Signal detection of statin-related DILI in age-stratified analysis.

Elderly individuals often have different degrees of liver function and kidney function decline, which may affect the metabolism of drugs and increase the risk of AE. Therefore, we divided statin cases into ≥65 years and <65 years groups for age-stratified analysis. Signals for six statins were detected in the ≥ 65 years group, including fluvastatin (ROR: 8.24, 95% CI: 6.62–10.27; IC: 2.85, 95% CI: 2.03–3.47), atorvastatin (ROR: 3.73, 95% CI: 3.56–3.90; IC: 1.79, 95% CI 1.64–1.94), lovastatin (ROR: 3.08, 95% CI: 2.04–4.65; IC: 1.56, 95% CI: 0.11–2.79), simvastatin (ROR: 2.96, 95% CI: 2.75–3.19; IC: 1.50, 95% CI: 1.25–1.75), pravastatin (ROR: 2.28, 95% CI: 1.88–2.76; IC: 1.15, 95% CI: 0.50–1.77), and rosuvastatin (ROR: 1.97, 95% CI: 1.81–2.15; IC: 0.95, 95% CI: 0.66–1.22) (Fig 2).

In the < 65 years old group, signals were detected for five drugs, including fluvastatin (ROR: 5.75, 95% CI: 4.38–7.56; IC: 2.38, 95% CI: 1.38–3.17), lovastatin (ROR: 3.32, 95% CI: 2.30–4.78; IC: 1.66, 95% CI: 0.37–2.75), pitavastatin (ROR: 2.98, 95% CI: 1.97–4.51; IC: 1.52, 95% CI: 0.06–2.75), atorvastatin (ROR: 2.64, 95% CI: 2.50–2.78; IC: 1.34, 95% CI 1.16–1.52), simvastatin (ROR: 2.43, 95% CI: 2.23–2.64; IC: 1.23, 95% CI: 0.94–1.51), rosuvastatin (ROR: 2.17, 95% CI: 2.00–2.34; IC: 1.08, 95% CI: 2.82–1.33), and pravastatin (ROR: 2.07, 95% CI: 1.65–2.59; IC: 1.02, 95% CI: 0.25–1.74) (Fig 2).

A stronger signal was detected in the ≥ 65 years group than in the group aged <65 years for simvastatin (ROR: 2.96 vs. 2.43), atorvastatin (ROR: 3.73 vs. 2.64), fluvastatin (ROR: 8.24 vs. 5.75), and pravastatin (ROR: 2.28 vs. 2.07).

### Onset time of adverse events

The onset time of DILI for each statins is shown in Table 3. The median time to onset of statin-related DILI was 26.0 days (IQR 7.0–80.0). The onset time of DILI was significantly different among different statins (*p* = 0.014). Significant differences were noted for atorvastatin vs. pitavastatin(*p* = 0.039), fluvastatin vs. pitavastatin(*p* = 0.007), fluvastatin vs. simvastatin (*p* = 0.031), pitavastatin vs. rosuvastatin(*p* = 0.012), and rosuvastatin vs. simvastatin (*p* = 0.028). Overall, the onset of statin-induced DILI occurred mainly within six months. During the initial stage of medication, more attention should be given to monitoring the changes in liver function indicators.

**Table 3:** Time to DILI onset reported by statins.

Drug name	DILI with time data reported (n)	Median time	IQR time(d)	P-values for DILI onset time among different statins (P < 0.05)
Atorvastatin	1256	24.5	7.0-69.0	vs. pitavastatin (*p* = 0.039)
Rosuvastatin	465	21	6.0-80.0	vs. simvastatin (*p* = 0.028), vs. pitavastatin (*p* = 0.012)
Simvastatin	301	35	11.0-113.0	vs. fluvastatin (*p* = 0.031), rosuvastatin (p=0.028)
Pravastatin	47	41	12.0-94.0	/
Fluvastatin	83	20	5.0-32.5	vs. simvastatin (*p*** =** 0.031), vs. pitavastatin(p=0.007)
Lovastatin	12	89	35.3-202.5	/
Pitavastatin	25	48	28.0-107.0	vs. atorvastatin (*p* = 0.039), vs. rosuvastatin (*p* = 0.012), vs. fluvastatin (p=0.007)
Cerivastatin	0	0	/	/

Note: This table only presented the results of the onset time of DILI among different statins with significant differences (P < 0.05). Those with P ≥ 0.05 were not listed.

## Discussion

To the best of our knowledge, this is the first largestcollection, comprehensive pharmacovigilance analysis of statin-related DILI based on the FAERS database. By analyzing 167,328 reports from the FAERS database, we identified 7,779 cases of statin-associated DILI. Our study revealed significant associations between hepatotoxicity and the following statins: fluvastatin, atorvastatin, simvastatin, lovastatin, rosuvastatin, and pravastatin. Furthermore, age was identified as a modifier of risk, with increasing age amplifying the severity of potential risks associated with lipophilic statins. These findings reevaluate and monitor the safety of statins, and may serve as a foundation and new insight for future investigations of statin-induced DILI, while facilitating medical safety.

Statins are fundamental drugs for the prevention and treatment of hypercholesterolemia and other cardiovascular diseases, which commonly affect hepatic chemistry, a phenomenon frequently observed in clinical practice. Reported DILI cases most frequently involved atorvastatin, rosuvastatin, and simvastatin, which accounted for 93.38% of all reported cases. From 2017 to 2023, the number of atorvastatin- and rosuvastatin-related DILI cases markedly increased by more than twofold, a trend potentially attributable to the growth in their clinical utilization in recent years. Our study found a slightly higher incidence in females than in males, consistent with findings from Sweden and Spain [27,37]. By contrast, the DILIN study revealed a clear female predominance [38]. Additionally, our study also found that the majority of statin-related DILI cases occurred in patients aged ≥65 years, which may be attributed to age-related changes in pharmacokinetics and frequent polypharmacy in the elderly [39]. Notably, statin-related DILI cases mainly occurred in Europe, North America, and Asia, with regional differences in specific statin reporting numbers. These differences may reflect varying prescription frequencies and suggest potential risk heterogeneity of specific drugs across populations, warranting further cross-ethnic genomic and clinical studies to clarify genetic influences on regional disparities.

Statin-induced liver injury exhibited a dose-dependent relationship, with inappropriate dosing as a key risk factor [40–42]. Jacobson et al. demonstrated that among patients receiving statin doses between 20–80 mg, alanine transaminase (ALT) levels increased progressively with higher dosages [40]. while Denus et al. reported that low-to-moderate doses of pravastatin, lovastatin, and simvastatin did not increase the risk of elevated liver enzymes compared with placebo [43]. In our study, a corresponding increase in DILI cases was observed as the daily atorvastatin dosage escalated from 10 mg/d to 80 mg/d. This observation highlights the need for cautious dose selection in clinical practice to mitigate DILI risk.

The pathogenesis of statin-induced DILI remains incompletely elucidated and may be associated with the intrinsic toxicity of the drugs, mitochondrial dysfunction, immunological factors, or reduced cholesterol levels [38,44–47]. In a French population-based prospective study, lipid-lowering drugs (12.5%) were among the most common causes of DILI, with 12% of DILI patients requiring hospitalization and 6% resulting in mortality [12]. In an Icelandic study, 3 of 96 enrolled patients (3.1%) developed hepatotoxicity associated with atorvastatin (2 cases) and simvastatin (1 case) [13]. In the Spanish Hepatotoxicity Registry, 18/461 (3%) patients were suspected of having DILI induced by lipid-lowering agents [16]. A report from the ALF research group revealed that 6 cases (4.5%) of DILI with acute liver failure were associated with the use of a statin medication [23]. As these previous studies have shown, statins seem to be the cause of liver injury and even acute liver failure. Nevertheless, most statin-induced DILI is self-limiting, often accompanied by mild to moderate elevations in transaminases, but rarely leads to chronic liver injury or death [48,49].

The necessity of liver function monitoring for statins is controversial and varies regionally. The initial FDA label recommended that clinicians check liver enzymes before starting statins, every six months, and after the dose increases [50]. In 2012, the FDA revised the label to cancel regular checks and monitor only at the beginning and when there are symptoms [51]. However, due to relatively high prevalence of chronic hepatitis B in China, doctors prefer regular liver function monitoring before, after, and during statin use for safety reasons. Furthermore, among patients administered with statins, the significant reduction in cardiovascular events largely outweighs the risk of these side effects [52,53]. However, as an increasing number of patients take statins, it is essential to monitor any side effects, conduct intensive research to identify susceptible patients, understand the mechanism of action, and manage these complications rationally to further enhance the safety of these drugs.

In our study, we identified eight statins reported to cause liver injury, observing significant associations with six of them. The strength of these signals was ranked as follows: fluvastatin > atorvastatin > simvastatin > lovastatin > rosuvastatin > pravastatin. Notably, the number of cases involving lovastatin and pitavastatin was relatively low, thus the findings for these drugs may be less precise. Cerivastatin was withdrawn from the market by the FDA in 2001 due to its severe rhabdomyolysis effects and the availability of better alternatives, thus the corresponding ROR in this study was reduced. Some studies have indicated that lipophilicity and extensive hepatic metabolism are indeed documented to generally increase the risk of hepatotoxicity [3,50]. Atorvastatin, fluvastatin, simvastatin, and lovastatin are lipophilic drugs, and their metabolism occurs mainly through cytochrome P-450, inhibitors of CYP450 enzymes leading to significant drug-drug interactions, and exhibit greater hepatotoxicity [50,54]. Whereas pravastatin, and pitavastatin are hydrophilic and undergo minimal metabolism by hepatic CYP enzymes, resulting in a markedly lower risk of acute liver injury and Rosuvastatin has intermediate behavior [50,55]. This may explain why most statin-induced liver injuries are caused by atorvastatin and simvastatin.

Advanced age may increase the risk of DILI. Our subgroup analysis revealed that among patients aged 65 years and older, the signals for fluvastatin, atorvastatin, and lovastatin were stronger than those for other statins. In the < 65 years old group, the signals for fluvastatin, lovastatin, and pitavastatin were stronger than those for the other statins. Notably, pitavastatin is a third-generation statin that is minimally metabolized by hepatic CYP enzymes and does not require dosage adjustment. However, our study indicates that pitavastatin poses a greater risk of DILI in the < 65-year-old group, which may be attributed to clinicians’ preference for prescribing pitavastatin in patients with a history of hepatotoxicity.

Regarding latency, the median latency of statin-related liver injury in our study varied significantly by drug type, ranging from 20.0 days (IQR 5.0–32.5 days) for fluvastatin to 89 days (IQR 35.3–202.5 days) for lovastatin. Previous studies have reported a median latency time of 155 days for statins in the DILIN study, which was longer than those observed in the Spain study(57 days) and Swedish study(90 days) [27,37,38]. Notably, an interesting phenomenon was the significant difference in DILI onset time across statin types. Of note, statin-related DILI predominantly occurs during the early phase of statin therapy, with 90.96% of cases developing within 6 months, consistent with a prior study [3]. Therefore, when prescribing statins, clinicians should pay attention to the occurrence of DILI, especially in the first 6 months.

Several studies have reported that fatal cases due to statin-related DILI are relatively rare, primarily associated with atorvastatin and simvastatin [27,56]. In the prospective DILIN study, statin-induced liver toxicity in 22 cases (1.85%), with most cases being mild to moderate liver injury, yet four cases were considered severe and one case with pre-existing alcoholic cirrhosis resulted in death [11]. In a large series study in Sweden, 8/747 (1%) of the cases were considered liver injury to be caused by statins, with two cases leading to death and one required liver transplantation, respectively [57]. The Spanish Hepatotoxicity separately noted two patient died but the deaths were not liver related [37]. We found that among spontaneous reports of DILI, the mortality rate of DILI caused by simvastatin was 12.15%, that of pravastatin was 9.09%, and that of atorvastatin was 8.56%, which were slightly higher than the mortality rates of DILI caused by fluvastatin (6.51%), rosuvastatin (6.29%), and lovastatin (5.88%). There are significant differences in mortality rates among different drugs. It is noteworthy that the FAERS database can be used as a pharmacovigilance tool and still fails to establish causality. The prognosis of DILI patients cannot be generalized but is specific to the individual circumstances, and is influenced by drug interactions, age, underlying diseases, genetic predispositions, and cost considerations [58]. However, we believe that early prediction will be more beneficial for prognosis.

Inevitably, this study has certain limitations. Firstly, FAERS is a spontaneous reporting database, may inevitably involve underreporting, missing data, and duplicate reports, such as the underlying causes of hospitalization and combination drugs. Secondly, as a retrospective study, all the signal detection results only indicate statistical associations between statins and DILI events, not causality or prevalence. Despite these limitations, the results of this comprehensive data-mining effort provide valuable insights into the safe and rational clinical application of statins based on large real-world sample study. Nevertheless, it is noteworthy that the number of reports of hepatotoxicity related to atorvastatin and rosuvastatin almost doubled after 2017 in this study, and further prospective studies are still needed to confirm the causal relationship.

Given the global prevalence of statin use and the potential for severe hepatotoxicity, clinicians should carefully assess patients’ liver function and implement early identification and preventive measures when prescribing statins, particularly lipophilic formulations or high-dose statins [3]. Additionally, adjuvant strategies to mitigate risks are worthy of consideration, such as repurposing drugs and giving various vitamins as D as prophylactic with immunomodulatory effect, which may exert positive impacts on liver diseases to be taken with statins [59].

## Conclusion

This study systematically evaluated the association between statins and hepatotoxicity in real-world practice. Overall, significant signals were observed between fluvastatin, atorvastatin, simvastatin, lovastatin, rosuvastatin, pravastatin and DILI, with hepatotoxicity predominantly occurring within 6 months of treatment initiation. Notably, the significant potential risk of DILI associated with lipophilic drugs such as fluvastatin, atorvastatin, and simvastatin increases with age. Additionally, a dose-dependent increase in atorvastatin-related hepatotoxicity was identified. These findings can be used to reevaluate and monitor the safety of statins, facilitating the development of strategies to mitigate drug adverse events.

## Supporting information

S1 Table Preferred terms for identifying drug-induced liver injury by SMQ (code: 20000007) narrow search in FAERS database. (DOCX)

S2 Table Algorithm for disproportionate analyses. (DOCX)

S3 Table Age analysis of DILI cases associated with statins in FAERS. (DOCX)

S4 Table Age analysis of Non-DILI cases associated with statins in FAERS. (DOCX)

S5 Table Sex analysis of DILI cases associated with statins in FAERS. (DOCX)

S6 Table Sex analysis of Non-DILI cases associated with statins in FAERS. (DOCX)

S7 Table Reporter of DILI cases associated with statins in FAERS. (DOCX)

S8 Table Reporter of Non-DILI cases associated with statins in FAERS. (DOCX)

S9 Table Reporter of DILI cases associated with different classes of statins in FAERS. (DOCX)

S10 Table Reporter of Non-DILI cases associated with different classes of statins in FAERS. (DOCX)

S11 Table Clinical outcomes of DILI cases associated with different dosages of atorvastatin in FAERS. (DOCX)

S12 Table Patient outcomes analysis of DILI cases associated with different classes of statins in FAERS. (DOCX)

S13 Table Patient outcomes analysis of Non-DILI cases associated with different classes of statins in FAERS. (DOCX)

## Abbreviations

ALF: acute liver failure
CVD: cardiovascular disease
DILI: drug-induced liver injury
FAERS: United States Food and Drug Administration Adverse Event Reporting System
