# Supplementary material for: Hepatotoxicity associated with statins: A retrospective pharmacovigilance study based on the FAERS database
Source: PLoS One. 2025 Jul 9;20(7):e0327500. doi: 10.1371/journal.pone.0327500 (PMC12240319; doi:10.1371/journal.pone.0327500)
Supplement: S1 Table — (DOCX) [file pone.0327500.s001.docx]

**S1 Table. Preferred terms for identifying drug-induced liver injury by SMQ (code: 20000007) narrow search in FAERS database.**

| **SMQ code** | **SMQ name** | **No.** | **PT code** | **Preferred term (PT)** |
| --- | --- | --- | --- | --- |
| 20000007 | Drug related hepatic disorders - severe events only (SMQ) | 1 | 10003827 | Autoimmune hepatitis |
|  |  | 2 | 10008909 | Chronic hepatitis |
|  |  | 3 | 10019717 | Hepatitis |
|  |  | 4 | 10019727 | Hepatitis acute |
|  |  | 5 | 10019754 | Hepatitis cholestatic |
|  |  | 6 | 10019755 | Hepatitis chronic active |
|  |  | 7 | 10019759 | Hepatitis chronic persistent |
|  |  | 8 | 10019772 | Hepatitis fulminant |
|  |  | 9 | 10019795 | Hepatitis toxic |
|  |  | 10 | 10023025 | Ischaemic hepatitis |
|  |  | 11 | 10029530 | Non-alcoholic fatty liver |
|  |  | 12 | 10049199 | Hepatic cytolysis |
|  |  | 13 | 10051015 | Radiation hepatitis |
|  |  | 14 | 10053219 | Non-alcoholic steatohepatitis |
|  |  | 15 | 10064676 | Graft versus host disease in liver |
|  |  | 16 | 10066263 | Acute graft versus host disease in liver |
|  |  | 17 | 10067737 | Lupus hepatitis |
|  |  | 18 | 10071198 | Allergic hepatitis |
|  |  | 19 | 10072160 | Chronic graft versus host disease in liver |
|  |  | 20 | 10076331 | Steatohepatitis |
|  |  | 21 | 10078962 | Immune-mediated hepatitis |
|  |  | 22 | 10080576 | Alloimmune hepatitis |
|  |  | 23 | 10019823 | Hepatoblastoma recurrent |
|  |  | 24 | 10027761 | Mixed hepatocellular cholangiocarcinoma |
|  |  | 25 | 10050842 | Liver carcinoma ruptured |
|  |  | 26 | 10055110 | Hepatic cancer metastatic |
|  |  | 27 | 10059318 | Hepatic cancer stage I |
|  |  | 28 | 10059319 | Hepatic cancer stage II |
|  |  | 29 | 10059324 | Hepatic cancer stage III |
|  |  | 30 | 10059325 | Hepatic cancer stage IV |
|  |  | 31 | 10062001 | Hepatoblastoma |
|  |  | 32 | 10067388 | Hepatic angiosarcoma |
|  |  | 33 | 10073069 | Hepatic cancer |
|  |  | 34 | 10073070 | Hepatic cancer recurrent |
|  |  | 35 | 10073071 | Hepatocellular carcinoma |
|  |  | 36 | 10073073 | Hepatobiliary cancer |
|  |  | 37 | 10073074 | Hepatobiliary cancer in situ |
|  |  | 38 | 10077861 | Cholangiosarcoma |
|  |  | 39 | 10085864 | Hepatic neuroendocrine tumour |
|  |  | 40 | 10086958 | Hepatic sarcoma |
|  |  | 41 | 10019695 | Hepatic neoplasm |
|  |  | 42 | 10061203 | Hepatobiliary neoplasm |
|  |  | 43 | 10004269 | Benign hepatic neoplasm |
|  |  | 44 | 10018821 | Haemangioma of liver |
|  |  | 45 | 10019629 | Hepatic adenoma |
|  |  | 46 | 10019646 | Hepatic cyst |
|  |  | 47 | 10052285 | Focal nodular hyperplasia |
|  |  | 48 | 10053973 | Hepatic cyst ruptured |
|  |  | 49 | 10054885 | Hepatic haemangioma rupture |
|  |  | 50 | 10067796 | Haemorrhagic hepatic cyst |
|  |  | 51 | 10077922 | Benign hepatobiliary neoplasm |
|  |  | 52 | 10079685 | Hepatic hamartoma |
|  |  | 53 | 10079889 | Hepatobiliary cyst |
|  |  | 54 | 10086088 | Hepatic lipoma |
|  |  | 55 | 10000804 | Acute hepatic failure |
|  |  | 56 | 10003445 | Ascites |
|  |  | 57 | 10003547 | Asterixis |
|  |  | 58 | 10004659 | Biliary cirrhosis |
|  |  | 59 | 10004664 | Biliary fibrosis |
|  |  | 60 | 10010075 | Coma hepatic |
|  |  | 61 | 10019637 | Hepatic atrophy |
|  |  | 62 | 10019641 | Hepatic cirrhosis |
|  |  | 63 | 10019660 | Hepatic encephalopathy |
|  |  | 64 | 10019663 | Hepatic failure |
|  |  | 65 | 10019668 | Hepatic fibrosis |
|  |  | 66 | 10019692 | Hepatic necrosis |
|  |  | 67 | 10019708 | Hepatic steatosis |
|  |  | 68 | 10019772 | Hepatitis fulminant |
|  |  | 69 | 10019837 | Hepatocellular injury |
|  |  | 70 | 10019845 | Hepatorenal failure |
|  |  | 71 | 10019846 | Hepatorenal syndrome |
|  |  | 72 | 10019851 | Hepatotoxicity |
|  |  | 73 | 10024670 | Liver disorder |
|  |  | 74 | 10024714 | Liver transplant |
|  |  | 75 | 10025129 | Lupoid hepatic cirrhosis |
|  |  | 76 | 10029530 | Non-alcoholic fatty liver |
|  |  | 77 | 10030210 | Oesophageal varices haemorrhage |
|  |  | 78 | 10036200 | Portal hypertension |
|  |  | 79 | 10039012 | Reye's syndrome |
|  |  | 80 | 10049199 | Hepatic cytolysis |
|  |  | 81 | 10049631 | Oedema due to hepatic disease |
|  |  | 82 | 10050897 | Portal hypertensive gastropathy |
|  |  | 83 | 10051010 | Duodenal varices |
|  |  | 84 | 10051012 | Gastric varices |
|  |  | 85 | 10051081 | Nodular regenerative hyperplasia |
|  |  | 86 | 10052274 | Hepatopulmonary syndrome |
|  |  | 87 | 10052279 | Renal and liver transplant |
|  |  | 88 | 10053219 | Non-alcoholic steatohepatitis |
|  |  | 89 | 10053244 | Hepatocellular foamy cell syndrome |
|  |  | 90 | 10056091 | Varices oesophageal |
|  |  | 91 | 10056956 | Subacute hepatic failure |
|  |  | 92 | 10057572 | Gastric varices haemorrhage |
|  |  | 93 | 10057573 | Chronic hepatic failure |
|  |  | 94 | 10061135 | Spontaneous bacterial peritonitis |
|  |  | 95 | 10061997 | Hepatectomy |
|  |  | 96 | 10061998 | Hepatic lesion |
|  |  | 97 | 10062000 | Hepatobiliary disease |
|  |  | 98 | 10062040 | Liver operation |
|  |  | 99 | 10063075 | Cryptogenic cirrhosis |
|  |  | 100 | 10064668 | Hepatic infiltration eosinophilic |
|  |  | 101 | 10065274 | Hepatic calcification |
|  |  | 102 | 10066597 | Gastrooesophageal variceal haemorrhage prophylaxis |
|  |  | 103 | 10066599 | Hepatic encephalopathy prophylaxis |
|  |  | 104 | 10066758 | Mixed liver injury |
|  |  | 105 | 10067125 | Liver injury |
|  |  | 106 | 10067281 | Portopulmonary hypertension |
|  |  | 107 | 10067338 | Retrograde portal vein flow |
|  |  | 108 | 10067365 | Hepatic hydrothorax |
|  |  | 109 | 10067823 | Splenic varices |
|  |  | 110 | 10067969 | Cholestatic liver injury |
|  |  | 111 | 10068547 | Bacterascites |
|  |  | 112 | 10068662 | Splenic varices haemorrhage |
|  |  | 113 | 10068923 | Portal hypertensive enteropathy |
|  |  | 114 | 10070815 | Acute yellow liver atrophy |
|  |  | 115 | 10070953 | Reynold's syndrome |
|  |  | 116 | 10071265 | Diabetic hepatopathy |
|  |  | 117 | 10071502 | Intestinal varices |
|  |  | 118 | 10072268 | Drug-induced liver injury |
|  |  | 119 | 10072284 | Varicose veins of abdominal wall |
|  |  | 120 | 10072319 | Gallbladder varices |
|  |  | 121 | 10073209 | Portal vein dilatation |
|  |  | 122 | 10073215 | Peripancreatic varices |
|  |  | 123 | 10073979 | Portal vein cavernous transformation |
|  |  | 124 | 10074726 | Portal fibrosis |
|  |  | 125 | 10076237 | Gastric variceal injection |
|  |  | 126 | 10076238 | Gastric variceal ligation |
|  |  | 127 | 10076331 | Steatohepatitis |
|  |  | 128 | 10076640 | Liver dialysis |
|  |  | 129 | 10077215 | Hepatic steato-fibrosis |
|  |  | 130 | 10077259 | Non-cirrhotic portal hypertension |
|  |  | 131 | 10077305 | Acute on chronic liver failure |
|  |  | 132 | 10078058 | Intestinal varices haemorrhage |
|  |  | 133 | 10078438 | White nipple sign |
|  |  | 134 | 10079446 | Portal hypertensive colopathy |
|  |  | 135 | 10080429 | Primary biliary cholangitis |
|  |  | 136 | 10080679 | Regenerative siderotic hepatic nodule |
|  |  | 137 | 10080860 | Acquired hepatocerebral degeneration |
|  |  | 138 | 10082480 | Cardiohepatic syndrome |
|  |  | 139 | 10083010 | Sugiura procedure |
|  |  | 140 | 10083406 | Immune-mediated cholangitis |
|  |  | 141 | 10083521 | Immune-mediated hepatic disorder |
|  |  | 142 | 10084797 | Flood syndrome |
|  |  | 143 | 10087030 | Omental oedema |
|  |  | 144 | 10087656 | Portal hypertensive biliopathy |
