# Supplementary material for: Hepatotoxicity associated with statins: A retrospective pharmacovigilance study based on the FAERS database
Source: PLoS One. 2025 Jul 9;20(7):e0327500. doi: 10.1371/journal.pone.0327500 (PMC12240319; doi:10.1371/journal.pone.0327500)
Supplement: S2 Table — (DOCX) [file pone.0327500.s002.docx]

**S2 Table. Algorithm for disproportionate analyses.**

| **Drugs** | **DILI event cases** | **All other adverse event cases** |
| --- | --- | --- |
| Target drug | a | b |
| All other drugs | c | d |
| ROR = $\frac{a/b}{\text{c}/d}$  95%CI for ROR = $e^{\text{ln(}\text{R}\text{OR)}\pm\text{1.96}\sqrt{(\frac{1}{a}+\frac{1}{b}+\frac{1}{c}+\frac{1}{d})}}$ | | |
| IC = log_2_$\frac{a(a+b+c+d)}{\text{(a+b)(a}+c)}$  E(IC) = log_2_$\frac{(a+\gamma11)(N+\alpha)(N+\beta)}{\text{(N+γ)(a}+b+\alpha1)(a+c+\beta1)}$  V(IC) ≈ ($\frac{1}{\text{log2}}$)^2^[$\frac{N-a+\gamma-\gamma11)}{\text{(a+γ11)(1}+N+\gamma)}+\frac{N-a-b+\alpha-\alpha1)}{\text{(a+b+α1)(1}+N+\alpha)}+\frac{N-a-c+\beta-\beta1)}{\text{(a+c+β1)(1}+N+\beta)}$]  γ = γ_11_$\frac{(N+\alpha)(N+\beta)}{\text{(a+b+α1)(a}+c+\beta1)}$  95%CI for IC = E(IC) ± 1.96$\sqrt{V(IC)}$  Where α=α_1_+α_2_, β=β_1_+β_2_, N=a+b+c+d, and the value of α_1_, α_2_, β_1_, β_2_ and γ_11_ were defined as 1. | | |

ROR: reporting odds ratio; IC: information component; 95%CI: 95% confidence interval.
