# Supplementary material for: Hepatotoxicity associated with statins: A retrospective pharmacovigilance study based on the FAERS database
Source: PLoS One. 2025 Jul 9;20(7):e0327500. doi: 10.1371/journal.pone.0327500 (PMC12240319; doi:10.1371/journal.pone.0327500)
Supplement: S3 Table — (DOCX) [file pone.0327500.s003.docx]

**S3 Table. Age analysis of DILI cases associated with statins in FAERS.**

| Drug/PT | <65 years |  | ＞65 years |  | Unkown |  |
| --- | --- | --- | --- | --- | --- | --- |
|  | DILI case number(n) | Proportion  (%) | DILI case number(n) | Proportion (%) | DILI case number(n) | Proportion (%) |
| Atorvastatin | 1408 | 33.85 | 2001 | 48.10 | 751 | 18.05 |
| Rosuvastatin | 665 | 41.82 | 568 | 35.72 | 357 | 22.45 |
| Simvastatin | 552 | 36.46 | 723 | 47.75 | 239 | 15.79 |
| Pravastatin | 79 | 35.91 | 110 | 50.00 | 31 | 14.09 |
| Fluvastatin | 58 | 34.32 | 93 | 55.03 | 18 | 10.65 |
| Lovastatin | 31 | 45.59 | 24 | 35.29 | 13 | 19.12 |
| Pitavastatin | 24 | 46.15 | 14 | 26.92 | 14 | 26.92 |
| Cerivastatin | 4 | 66.67 | 1 | 16.67 | 1 | 16.67 |
