# Supplementary material for: Hepatotoxicity associated with statins: A retrospective pharmacovigilance study based on the FAERS database
Source: PLoS One. 2025 Jul 9;20(7):e0327500. doi: 10.1371/journal.pone.0327500 (PMC12240319; doi:10.1371/journal.pone.0327500)
Supplement: S4 Table — (DOCX) [file pone.0327500.s004.docx]

**S4 Table. Age analysis of Non-DILI cases associated with statins in FAERS.**

| Drug/PT | <65 years |  | ＞65 years |  | Unkown |  |
| --- | --- | --- | --- | --- | --- | --- |
|  | Non-DILI case number(n) | Proportion  (%) | Non-DILI case number(n) | Proportion (%) | Non-DILI case number(n) | Proportion (%) |
| Atorvastatin | 24028 | 30.58 | 26993 | 34.36 | 27541 | 35.06 |
| Rosuvastatin | 13758 | 33.97 | 14236 | 35.15 | 12507 | 30.88 |
| Simvastatin | 10201 | 34.38 | 12102 | 40.79 | 7367 | 24.83 |
| Pravastatin | 1708 | 27.81 | 2375 | 38.67 | 2059 | 33.52 |
| Fluvastatin | 451 | 31.87 | 556 | 39.29 | 408 | 28.83 |
| Lovastatin | 418 | 29.46 | 384 | 27.06 | 617 | 43.48 |
| Pitavastatin | 360 | 22.53 | 459 | 28.72 | 779 | 48.75 |
| Cerivastatin | 11 | 42.31 | 10 | 38.46 | 5 | 19.23 |
