# Supplementary material for: Hepatotoxicity associated with statins: A retrospective pharmacovigilance study based on the FAERS database
Source: PLoS One. 2025 Jul 9;20(7):e0327500. doi: 10.1371/journal.pone.0327500 (PMC12240319; doi:10.1371/journal.pone.0327500)
Supplement: S5 Table — (DOCX) [file pone.0327500.s005.docx]

**S5 Table. Sex analysis of DILI cases associated with statins in FAERS.**

| Drug/PT | Famale |  | Male |  | Unkown |  |
| --- | --- | --- | --- | --- | --- | --- |
|  | DILI case number(n) | Proportion  (%) | DILI case number(n) | Proportion (%) | DILI case number(n) | Proportion (%) |
| Atorvastatin | 2132 | 51.25 | 1737 | 41.75% | 291 | 7.00 |
| Rosuvastatin | 729 | 45.85 | 733 | 46.10% | 128 | 8.05 |
| Simvastatin | 661 | 43.66 | 733 | 48.41% | 120 | 7.93 |
| Pravastatin | 112 | 50.91 | 88 | 40.00% | 20 | 9.09 |
| Fluvastatin | 107 | 63.31 | 55 | 32.54% | 7 | 4.14 |
| Lovastatin | 34 | 50.00 | 32 | 47.06% | 2 | 2.94 |
| Pitavastatin | 18 | 34.62 | 22 | 42.31% | 12 | 23.08 |
| Cerivastatin | 3 | 50.00 | 3 | 50.00% | 0 | 0.00 |
