# Supplementary material for: Hepatotoxicity associated with statins: A retrospective pharmacovigilance study based on the FAERS database
Source: PLoS One. 2025 Jul 9;20(7):e0327500. doi: 10.1371/journal.pone.0327500 (PMC12240319; doi:10.1371/journal.pone.0327500)
Supplement: S6 Table — (DOCX) [file pone.0327500.s006.docx]

**S6 Table. Sex analysis of Non-DILI cases associated with statins in FAERS.**

| Drug/PT | Famale |  | Male |  | Unkown |  |
| --- | --- | --- | --- | --- | --- | --- |
|  | Non-DILI case number(n) | Proportion  (%) | Non-DILI case number(n) | Proportion (%) | Non-DILI case number(n) | Proportion (%) |
| Atorvastatin | 37853 | 48.18 | 31196 | 39.71 | 9513 | 12.11 |
| Rosuvastatin | 20766 | 51.27 | 16601 | 40.99 | 3134 | 7.74 |
| Simvastatin | 12362 | 41.66 | 15001 | 50.56 | 2307 | 7.78 |
| Pravastatin | 2879 | 46.87 | 2565 | 41.76 | 698 | 11.36 |
| Lovastatin | 666 | 46.93 | 626 | 44.12 | 127 | 8.95 |
| Fluvastatin | 659 | 46.57 | 679 | 47.99 | 77 | 5.44 |
| Pitavastatin | 573 | 35.86 | 427 | 26.72 | 598 | 37.42 |
| Cerivastatin | 6 | 23.08 | 18 | 69.23 | 2 | 7.69 |
