# Supplementary material for: Hepatotoxicity associated with statins: A retrospective pharmacovigilance study based on the FAERS database
Source: PLoS One. 2025 Jul 9;20(7):e0327500. doi: 10.1371/journal.pone.0327500 (PMC12240319; doi:10.1371/journal.pone.0327500)
Supplement: S7 Table — (DOCX) [file pone.0327500.s007.docx]

**S7 Table. Reporter of DILI cases associated with statins in FAERS.**

| Drug/PT | Health-professional |  | Non-health professionals |  | Unknown |  |
| --- | --- | --- | --- | --- | --- | --- |
|  | DILI case number(n) | Proportion(%) | DILI case number(n) | Proportion (%) | DILI case number(n) | Proportion (%) |
| Atorvastatin | 3248 | 78.08 | 746 | 17.93 | 166 | 3.99 |
| Rosuvastatin | 1129 | 71.01 | 247 | 15.53 | 214 | 13.46 |
| Simvastatin | 1204 | 79.52 | 209 | 13.80 | 101 | 6.67 |
| Pravastatin | 177 | 80.45 | 39 | 17.73 | 4 | 1.82 |
| Fluvastatin | 136 | 80.47 | 14 | 8.28 | 19 | 11.24 |
| Lovastatin | 48 | 70.59 | 13 | 19.12 | 7 | 10.29 |
| Pitavastatin | 43 | 82.69 | 6 | 11.54 | 3 | 5.77 |
| Cerivastatin | 2 | 33.33 | 0 | 0.00 | 4 | 66.67 |
