# Supplementary material for: Hepatotoxicity associated with statins: A retrospective pharmacovigilance study based on the FAERS database
Source: PLoS One. 2025 Jul 9;20(7):e0327500. doi: 10.1371/journal.pone.0327500 (PMC12240319; doi:10.1371/journal.pone.0327500)
Supplement: S8 Table — (DOCX) [file pone.0327500.s008.docx]

**S8 Table. Reporter of Non-DILI cases associated with statins in FAERS.**

| Drug/PT | Health-professional | | Non-health professionals | | Unknown | |
| --- | --- | --- | --- | --- | --- | --- |
|  | Non-DILI case number(n) | Proportion  (%) | Non-DILI case number(n) | Proportion (%) | Non-DILI case number(n) | Proportion (%) |
| Atorvastatin | 33025 | 42.04 | 41597 | 52.95 | 3940 | 5.02 |
| Rosuvastatin | 16103 | 39.76 | 16156 | 39.89 | 8242 | 20.35 |
| Simvastatin | 17889 | 60.29 | 8403 | 28.32 | 3378 | 11.39 |
| Pravastatin | 3030 | 49.33 | 2684 | 43.70 | 428 | 6.97 |
| Fluvastatin | 951 | 67.21 | 323 | 22.83 | 141 | 9.96 |
| Lovastatin | 574 | 40.45 | 666 | 46.93 | 179 | 12.61 |
| Pitavastatin | 908 | 56.82 | 583 | 36.48 | 107 | 6.70 |
| Cerivastatin | 6 | 23.08 | 4 | 15.38 | 16 | 61.54 |
