# Supplementary material for: Hepatotoxicity associated with statins: A retrospective pharmacovigilance study based on the FAERS database
Source: PLoS One. 2025 Jul 9;20(7):e0327500. doi: 10.1371/journal.pone.0327500 (PMC12240319; doi:10.1371/journal.pone.0327500)
Supplement: S9 Table — (DOCX) [file pone.0327500.s009.docx]

**S9 Table. Reporter of DILI cases associated with different classes of statins in FAERS.**

| Drug/PT | North America | | Europe | | Asian | | Oceania | | South America |  | Africa |  | Unspecified |  |
| --- | --- | --- | --- | --- | --- | --- | --- | --- | --- | --- | --- | --- | --- | --- |
|  | DILI case number(n) | Proportion  (%) | DILI case number(n) | Proportion (%) | DILI case number(n) | Proportion (%) | DILI case number(n) | Proportion (%) | DILI case number(n) | Proportion (%) | DILI case number(n) | Proportion (%) | DILI case number(n) |  |
| Atorvastatin | 1008 | 24.23 | 2300 | 55.29 | 430 | 10.34 | 60 | 1.44 | 76 | 1.83 | 18 | 0.43 | 268 | 6.44 |
| Rosuvastatin | 624 | 39.25 | 457 | 28.74 | 378 | 23.77 | 22 | 1.38 | 14 | 0.88 | 9 | 0.57 | 86 | 5.41 |
| Simvastatin | 416 | 27.48 | 845 | 55.81 | 74 | 4.89 | 21 | 1.39 | 22 | 1.45 | 6 | 0.40 | 130 | 8.59 |
| Pravastatin | 51 | 23.18 | 126 | 57.27 | 14 | 6.36 | 2 | 0.91 | 0 | 0.00 | 0 | 0.00 | 27 | 12.27 |
| Fluvastatin | 13 | 7.69 | 62 | 36.69 | 73 | 43.20 | 0 | 0.00 | 4 | 2.37 | 0 | 0.00 | 17 | 10.06 |
| Lovastatin | 45 | 66.18 | 11 | 16.18 | 3 | 4.41 | 0 | 0.00 | 0 | 0.00 | 0 | 0.00 | 9 | 13.24 |
| Pitavastatin | 7 | 13.46 | 16 | 30.77 | 28 | 53.85 | 0 | 0.00 | 0 | 0.00 | 0 | 0.00 | 1 | 1.92 |
| Cerivastatin | 1 | 16.67 | 1 | 16.67 | 0 | 0.00 | 0 | 0.00 | 0 | 0.00 | 0 | 0.00 | 4 | 66.67 |
