# Supplementary material for: Hepatotoxicity associated with statins: A retrospective pharmacovigilance study based on the FAERS database
Source: PLoS One. 2025 Jul 9;20(7):e0327500. doi: 10.1371/journal.pone.0327500 (PMC12240319; doi:10.1371/journal.pone.0327500)
Supplement: S10 Table — (DOCX) [file pone.0327500.s010.docx]

**S10 Table. Reporter of Non-DILI cases associated with different classes of statins in FAERS.**

| Drug/PT | North America | | Europe | | Asian | | Oceania | | South America | | Africa | | Unspecified |  |
| --- | --- | --- | --- | --- | --- | --- | --- | --- | --- | --- | --- | --- | --- | --- |
|  | Non-DILI case number(n) | Proportion  (%) | Non-DILI case number(n) | Proportion (%) | Non-DILI case number(n) | Proportion (%) | Non-DILI case number(n) | Proportion (%) | Non-DILI case number(n) | Proportion (%) | Non-DILI case number(n) | Proportion (%) | Non-DILI case number(n) | Proportion (%) |
| Atorvastatin | 47407 | 60.34 | 18456 | 23.49 | 3754 | 4.78 | 974 | 1.24 | 4562 | 5.81 | 417 | 0.53 | 2992 | 3.81 |
| Rosuvastatin | 27495 | 67.89 | 5989 | 14.79 | 1512 | 3.73 | 483 | 1.19 | 911 | 2.25 | 125 | 0.31 | 3986 | 9.84 |
| Simvastatin | 10641 | 35.86 | 15339 | 51.70 | 464 | 1.56 | 372 | 1.25 | 375 | 1.26 | 66 | 0.22 | 2413 | 8.13 |
| Pravastatin | 3146 | 51.22 | 2206 | 35.92 | 156 | 2.54 | 39 | 0.63 | 2 | 0.03 | 3 | 0.05 | 590 | 9.61 |
| Fluvastatin | 256 | 18.09 | 511 | 36.11 | 302 | 21.34 | 4 | 0.28 | 43 | 3.04 | 9 | 0.64 | 290 | 20.49 |
| Lovastatin | 1210 | 85.27 | 41 | 2.89 | 8 | 0.56 | 0 | 0.00 | 5 | 0.35 | 0 | 0.00 | 155 | 10.92 |
| Pitavastatin | 1086 | 67.96 | 126 | 7.88 | 356 | 22.28 | 0 | 0.00 | 15 | 0.94 | 0 | 0.00 | 15 | 0.94 |
| Cerivastatin | 8 | 30.77 | 4 | 15.38 | 0 | 0.00 | 0 | 0.00 | 0 | 0.00 | 0 | 0.00 | 14 | 53.85 |
