# Supplementary material for: Hepatotoxicity associated with statins: A retrospective pharmacovigilance study based on the FAERS database
Source: PLoS One. 2025 Jul 9;20(7):e0327500. doi: 10.1371/journal.pone.0327500 (PMC12240319; doi:10.1371/journal.pone.0327500)
Supplement: S11 Table — (DOCX) [file pone.0327500.s011.docx]

**S11 Table. Clinical outcomes of DILI cases associated with different dosages of atorvastatin in FAERS.**

| **Drug/PT** | **DILI Case number (n)** | **Death**  **(%)** | **Life-Threatening**  **(%)** | **Hospitalization**  **(%)** | **Disability**  **(%)** | **Required Intervention**  **(%)** | **Congenital Anomaly(%)** | **Other Serious(%)** | **Unkown(%)** |
| --- | --- | --- | --- | --- | --- | --- | --- | --- | --- |
| 5mg/d Atorvastatin | 45 | 2(4.44) | 2(4.44) | 30(66.67) | 1(2.22) | 0(0.00) | 0(0.00) | 9(20.00) | 1(2.22) |
| 10mg/d Atorvastatin | 471 | 51(10.83) | 28(5.94) | 184(39.07) | 12(2.55) | 1(0.21) | 0(0.00) | 184(39.07) | 11(2.34) |
| 20mg/d Atorvastatin | 564 | 38(6.74) | 41(7.27) | 220(46.51) | 18(3.19) | 0(0.00) | 0(0.00) | 237(42.02) | 10(1.77) |
| 40mg/d Atorvastatin | 559 | 46(8.23) | 48(8.59) | 260(46.51) | 3(0.54) | 1(0.18) | 0(0.00) | 194(34.70) | 7(1.25) |
| 80mg/d Atorvastatin | 655 | 50(7.63) | 32(4.89) | 374(57.10) | 5(0.76) | 0(0.00) | 0(0.00) | 189(28.85) | 5(0.76) |
|  |  | *P*=0.123 | *P*=0.001 | *P*=0.001 | *P*=0.001 | *P*=0.565 | / | *P*=0.001 | *P*=0.133 |
