# Supplementary material for: Hepatotoxicity associated with statins: A retrospective pharmacovigilance study based on the FAERS database
Source: PLoS One. 2025 Jul 9;20(7):e0327500. doi: 10.1371/journal.pone.0327500 (PMC12240319; doi:10.1371/journal.pone.0327500)
Supplement: S12 Table — (DOCX) [file pone.0327500.s012.docx]

**S12 Table. Patient outcomes analysis of DILI cases associated with different classes of statins in FAERS.**

| Drug/PT | Death (%) | Life-Threatening (%) | Hospitalization (%) | Disability  (%) | Required Intervention(%) | Congenital Anomaly(%) | Other Serious (%) | Unkown (%) |
| --- | --- | --- | --- | --- | --- | --- | --- | --- |
| Atorvastatin* | 356(8.56%) | 245(5.89%) | 1742(41.88%) | 67(1.61%) | 8(0.19%) | 1(0.02%) | 1601(38.49%) | 140(3.37%) |
| Rosuvastatin* | 100(6.29%) | 104(6.54%) | 533(33.52%) | 30(1.89%) | 29(1.82%) | 1(0.06%) | 635(39.94%) | 158(9.94%) |
| Simvastatin* | 184(12.15%) | 207(13.67%) | 622(41.08%) | 31(2.05%) | 5(0.33%) | 0(0.00%) | 423(27.94%) | 42(2.77%) |
| Pravastatin* | 20(9.09%) | 7(3.18%) | 94(42.73%) | 3(1.36%) | 0(0.00%) | 0(0.00%) | 86(39.09%) | 10(4.55%) |
| Fluvastatin* | 11(6.51%) | 12(7.10%) | 66(39.05%) | 7(4.14%) | 1(0.59%) | 0(0.00%) | 70(41.42%) | 2(1.18%) |
| Lovastatin* | 4(5.88%) | 6(8.82%) | 26(38.24%) | 3(4.41%) | 2(2.94%) | 0(0.00%) | 19(27.94%) | 8(11.76%) |
| Pitavastatin | 4(7.69%) | 6(11.54%) | 23(44.23%) | 0(0.00%) | 0(0.00%) | 0(0.00%) | 17(32.69%) | 2(3.85%) |
| Cerivastatin | 0(0.00%) | 0(0.00%) | 2(33.33%) | 3(50.00%) | 0(0.00%) | 0(0.00%) | 1(16.67%) | 0(0.00%) |
