# Supplementary material for: Hepatotoxicity associated with statins: A retrospective pharmacovigilance study based on the FAERS database
Source: PLoS One. 2025 Jul 9;20(7):e0327500. doi: 10.1371/journal.pone.0327500 (PMC12240319; doi:10.1371/journal.pone.0327500)
Supplement: S13 Table — (DOCX) [file pone.0327500.s013.docx]

**S13 Table. Patient outcomes analysis of Non-DILI cases associated with different classes of statins in FAERS.**

| Drug/PT | Death (%) | Life-Threatening (%) | Hospitalization (%) | Disability(%) | Required Intervention(%) | Congenital Anomaly(%) | Other Serious (%) | Unkown (%) |
| --- | --- | --- | --- | --- | --- | --- | --- | --- |
| Atorvastatin* | 4459(5.68%) | 1887(2.40%) | 12245(15.59%) | 3221(4.10%) | 276(0.35%) | 85(0.11%) | 36229(46.12%) | 20160(25.66%) |
| Rosuvastatin* | 982(2.42%) | 1055(2.60%) | 6410(15.83%) | 1413(3.49%) | 582(1.44%) | 25(0.06%) | 14594(36.03%) | 15440(38.12%) |
| Simvastatin* | 1332(4.49%) | 2173(7.32%) | 7783(26.23%) | 3417(11.52%) | 330(1.11%) | 71(0.24%) | 10217(34.44%) | 4347(14.65%) |
| Pravastatin* | 254(4.14%) | 263(4.28%) | 1049(17.08%) | 324(5.28%) | 39(0.63%) | 15(0.24%) | 1871(30.46%) | 2327(37.89%) |
| Fluvastatin* | 90(6.36%) | 70(4.95%) | 423(29.89%) | 57(4.03%) | 8(0.57%) | 1(0.07%) | 650(45.92%) | 116(8.20%) |
| Lovastatin* | 45(3.17%) | 74(5.21%) | 203(14.31%) | 78(5.50%) | 43(3.03%) | 14(0.99%) | 288(20.30%) | 674(47.50%) |
| Pitavastatin | 56(3.50%) | 31(1.94%) | 283(17.71%) | 45(2.82%) | 7(0.44%) | 1(0.06%) | 396(24.78%) | 779(48.75%) |
| Cerivastatin | 2(7.69%) | 3(11.54%) | 5(19.23%) | 6(23.08%) | 0(0.00%） | 0(0.00%) | 7(26.92%) | 3(11.54%) |
